# A new dual-thickness semi-transparent beamstop for small-angle X-ray scattering

**DOI:** 10.1107/S1600577524007392

**Published:** 2024-08-25

**Authors:** Haijuan Wu, Zhihong Li

**Affiliations:** a Beijing Synchrotron Radiation Facility, Institute of High Energy Physics Chinese Academy of Sciences Beijing100049 People’s Republic of China; b College of Nuclear Science and Technology University of Chinese Academy of Sciences Beijing100049 People’s Republic of China; ESRF – The European Synchrotron, France

**Keywords:** SAXS, transmission beam, semi-transparent beamstop, gradient attenuation, background subtraction

## Abstract

An innovative dual-thickness semi-transparent beamstop designed to enhance the performance of small-angle X-ray scattering (SAXS) experiments is introduced. This design integrates two absorbers of differing thicknesses side by side into a single attenuator, known as a beamstop. Instead of completely stopping the direct beam, it attenuates it, allowing the SAXS detector to measure the transmitted beam through the sample. This approach achieves true synchronization in measuring both scattered and transmitted signals and effectively eliminates higher-order harmonic contributions when determining the transmission light intensity through the sample. This facilitates and optimizes signal detection and background subtraction. This contribution details the theoretical basis and practical implementation of this solution at the SAXS station on the 1W2A beamline at the Beijing Synchrotron Radiation Facility. It also anticipates its application at other SAXS stations, including that at the forthcoming High Energy Photon Source, providing an effective solution for high-precision SAXS experiments.

## Abstract 2

A novel method for transmission intensity measurement and scattering intensity normalization is developed. This method enables true synchronization of scattering and transmission signal measurements, and separation of the transmission intensity of fundamental radiation from the attenuated direct beam.

## 1. Introduction

### 1.1. SAXS

Small-angle X-ray scattering (SAXS) is a powerful technique used to study the structure of materials at the nanometre scale (Glatter & Kratky, 1982 ▸; Gille, 2013 ▸; Li *et al.*, 2016 ▸). Various brands and styles of SAXS instruments based on laboratory light sources are available on the market. However, with the advancement of synchrotron radiation facilities, SAXS experimental stations utilizing synchrotron radiation with high intensity and excellent collimation as X-ray sources have become the primary experimental base for SAXS. This development has significantly enhanced the temporal and spatial resolution of SAXS experiments (Tsakanov, 2022 ▸; Brosey & Tainer, 2019 ▸).

The main components of a SAXS instrument include a light source, optical system, sample stage, vacuum tube and detector (Schnablegger & Singh, 2017 ▸; Polizzi & Spinozzi, 2015 ▸; Jeffries *et al.*, 2021 ▸). X-rays emitted by the light source are monochromated, focused or collimated by the optical elements before irradiating the sample. When the incident X-rays pass through the sample, various phenomena such as absorption, transmission and scattering occur. Typically, the transmitted X-rays pass through the camera vacuum tube and are blocked by a beamstop, while the scattered X-rays within a small angle range (usually 2θ ≤ 5°) pass through the vacuum tube and are detected by the SAXS detector (Feigin & Svergun, 1987 ▸). The intensity of both the transmitted and scattered X-rays depends on the energy and intensity of the incident X-rays, as well as the composition and thickness of the sample (Glatter & Kratky, 1982 ▸).

### 1.2. Background subtraction

In SAXS experiments, the scattering signals collected during sample measurement typically include the scattering from both the sample itself and the background. To isolate the scattering intensity of the pure sample, it is necessary to measure both the sample and the background under identical conditions (Lindner & Zemb, 1991 ▸; Pauw, 2014 ▸), and then subtract the background, as shown in equation (1) (Feigin & Svergun, 1987 ▸),
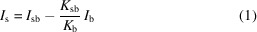
where *I* refers to the scattering intensity; *K* refers to the transmission beam intensity; and the subscripts s, b and sb refer to the pure sample, the background and a mixture of sample and background, respectively. The ratio *K*_sb_/*K*_b_ in equation (1) can be called the normalization factor, which corrects for inconsistencies in experimental conditions (such as incident beam intensity, exposure time, absorption, *etc*.) when measuring the sample and the background. In most cases, its value is not equal to 1. This background subtraction process is also referred to as normalization by some researchers. It can be seen from equation (1) that the measurement of transmission beam intensity directly affects the accuracy of background subtraction.

### 1.3. Conventional approaches

Different detectors in SAXS instruments have varying tolerance levels for light intensity. For instance, the photon-counting detector Pilatus 1MF at the SAXS station on the 1W2A beamline at Beijing Synchrotron Radiation Facility (BSRF) has a maximum count rate of 2 × 10^6^ counts pixel^−1^ s^−1^ and a count cutoff of 1280469 counts. In practice, if the transmission light is too strong, as is often the case with many synchrotron radiation beams, it cannot directly irradiate the SAXS detector. Therefore, a beamstop is necessary to block or attenuate the transmission beam to protect the detector and improve the signal-to-noise ratio (Kumar, 2016 ▸; San Emeterio *et al.*, 2022 ▸; Koch *et al.*, 2003 ▸). Beamstops made of high-*Z* metals (lead, tungsten, tantalum, *etc*.) are generally placed at the end of the camera vacuum tube near the detector.

Historically, ionization chambers were used for measuring transmission light intensity in SAXS (Sarvestani *et al.*, 1998 ▸; Menk *et al.*, 2000 ▸). These chambers, which have a larger outer volume and a smaller X-ray transmission aperture, have limitations when used in a vacuum. They need to be placed downstream and close to the sample to avoid affecting the scattering angle, but they receive both transmission and scattering signals, leading to background subtraction errors. With the increase in beam flux and the shortening of exposure times, ionization chambers have become less common.

Currently, photodiodes are more popular for this purpose. Photodiodes are relatively small and can be integrated with the beamstop, allowing for direct detection of transmission signals (Haas *et al.*, 2023 ▸; Li *et al.*, 2014 ▸; Owen *et al.*, 2009 ▸; Ellis *et al.*, 2003 ▸; Narayanan *et al.*, 2001 ▸) or indirect detection through back-scattered signals (Blanchet *et al.*, 2015 ▸). The former has limitations in size and lifetime, while the latter is too sensitive to beam position and lacks uniform response in space. Similar to many other SAXS stations, a photodiode integrated into the beamstop is used to measure the transmission beam intensity at the SAXS station on the 1W2A beamline at the BSRF (Li *et al.*, 2014 ▸).

In addition, another method involves using single-crystal chemical vapor deposition (CVD) diamond sensors to detect the intensity of transmitted direct beams. Similar to ionization chambers and photodiodes, this method also involves the photoelectric effect and the amplification and reading of weak electrical signals. It can be used in a vacuum, but its current applications are limited (Desjardins *et al.*, 2021 ▸).

When using either an ionization chamber or a photodiode, transmitted and scattered X-rays are measured by different detectors, which have different counting rates, response speeds and detection efficiencies. This can lead to systematic errors in intensity measurements. Additionally, the control signal logic and circuitry are complex, making true synchronization between the detection of transmission and scattering X-rays challenging. This can cause errors in intensity measurement, especially when the signal is weak or the exposure time is short. For example, when the exposure time is as short as 4 ms, the Pilatus 1MF detector at the SAXS station on the 1W2A beamline at BSRF can respond normally, but the photodiode does not. This may be due to limitations in the sensitivity of the photodiode or its amplification readout system.

In recent years, a method has emerged that uses a ‘semi-transparent’ beamstop combined with the SAXS detector (Lyngsø & Pedersen, 2021 ▸) to detect the intensity of transmitted direct beams. The semi-transparent beamstop is made of a thin metal absorber that attenuates but does not stop the transmission beam, allowing it to be measured by the SAXS detector. This enables simultaneous detection of transmission and scattering X-rays using the same detector. However, higher-order harmonics may significantly affect the attenuated signals detected by the detector, which have been neglected by existing solutions, causing certain errors.

### 1.4. New approach

To address this issue, this study develops a new dual-thickness semi-transparent beamstop that combines two absorbers of different thicknesses. This design functions as a ‘semi-transparent’ beamstop to facilitate the detection of transmission intensity and background subtraction from scattering intensity. The key to this solution is transforming the traditional beamstop into a beam attenuator with gradient attenuation sheets. This allows for the simultaneous measurement of both transmission and scattering X-rays using the same detector while eliminating the contribution of higher-order harmonics in deriving the transmitted light intensity. Consequently, this optimizes the performance of SAXS experiments. This contribution presents the theory, testing and prospects of this new solution.

## 2. Theory

### 2.1. Formula derivation

It is well known that SAXS typically requires the incident X-rays to be as pure as possible, with a high fundamental radiation content and low higher-order harmonic content to improve spectral quality. The nature of the light source and optical elements determines that the incident beam is composed of harmonics with different energies and fluxes (Raimondi *et al.*, 2023 ▸; Elleaume & Ropert, 2003 ▸; Jaeschke *et al.*, 2016 ▸; Li *et al.*, 2021 ▸). Besides the fundamental radiation (first harmonic), there is also a certain proportion of higher-order harmonics. These higher-order harmonics can be routinely suppressed using components such as insertion devices, monochromators and mirrors, but they cannot be eliminated (Chen *et al.*, 2018 ▸; Polikarpov *et al.*, 2014 ▸; Pauwels & Douissard, 2022 ▸). The final ratio between different harmonics in the beam can be determined by theoretical calculation based on the parameters of the light source and optical elements or by experimental measurement.

Suppose the total number of main harmonics to be considered in the incident beam is *n* (fundamental radiation + higher-order harmonics). The transmission beam intensity passing through a sample (including background) can be approximately represented as the sum of the intensity of each harmonic passing through the sample (including background), as shown in equation (2),
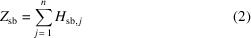
where *Z*_sb_ is the total intensity of the transmission beam passing through the sample (including background); *j* is the harmonic serial number (*j* = 1–*n*) in the beam; and *H*_sb,*j*_ is the intensity of the *j*th serial harmonic passing through the sample (including background).

The core of the new approach is to use absorber sheets to attenuate the transmission beam so that it can be measured by the scattering signal detector. According to the law of light absorption, the direct beam intensity *D*_sb_ received by the detector after passing through the sample (including background) and the absorber can be expressed by equation (3),
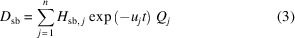
where μ_*j*_ is the absorption coefficient of the absorber to the *j*th serial harmonic; *t* is the absorber sheet thickness; and *Q*_*j*_ is the detection efficiency of the SAXS detector for the *j*th serial harmonic.

*H*_sb,*j*_ in equation (3) can be expressed as equation (4),
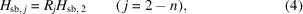
where, *H*_sb,2_ is the intensity of the second serial harmonic (*j* = 2) after passing through the sample (including the background), and *R*_*j*_ is the flux ratio of the higher-order harmonic (*j* = 2–*n*) to the second serial harmonic (*j* = 2) after passing through the sample (including background).

In theory, this equation is universal, but determining the coefficient *R*_*j*_ is limited by the X-ray energy and the sample. It is impractical to measure *R*_*j*_ of each sample during every measurement. Fortunately, in SAXS, samples studied using fundamental radiation usually have very high transmittance to higher-order harmonics. That is, compared with the fundamental radiation, the higher-order harmonic flux changes little before and after passing through the sample. Therefore, it can be assumed that the coefficient *R*_*j*_ is approximately equal in the incident and transmitted beam, which can be approximated as fixed and does not need to be measured or calculated for every sample.

Equation (5) can be obtained from equations (3) and (4),
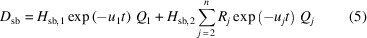
where *H*_sb,1_ is the intensity of the fundamental radiation (*j* = 1) after passing through the sample (including the background).

In equation (5), there are two unknowns, *H*_sb,1_ and *H*_sb,2_. Therefore, it is necessary to measure the absorber sheets of two thicknesses and solve the corresponding set of equations. The intensity of the transmission beam obtained from the SAXS detector through the sample (including the background) and *i* serial absorber sheet with thickness *t*_*i*_ can be represented by *D*_sb,*i*_ and expressed by equation (6),
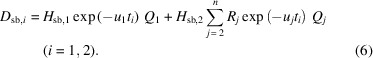
Similarly, the intensity of the transmission beam obtained from the SAXS detector through the background and *i* serial absorber sheet with thickness *t*_*i*_ can be represented by *D*_b,*i*_ and expressed by equation (7),
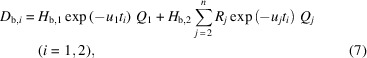
where *H*_b,*j*_ is the intensity of the *j*th serial harmonic after passing through the background.

The required normalization factor *K*_sb_/*K*_b_ in equation (1) can be solved from equations (6) and (7), as shown in (8),
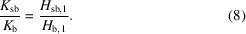
The background subtraction of scattering data can then be completed by substituting the normalized factor *K*_sb_/*K*_b_ into equation (1).

### 2.2. Design rule

The selection of the material, thickness and size of the absorber requires comprehensive consideration of several factors, including elemental composition, transmittance, beam size and parasitic scattering, following some basic rules.

The absorber material should be a high-purity metal or compound with a known absorption coefficient, good stability in temperature and time, and an appropriate absorption edge beyond the beam energy.

The thickness of the absorber depends primarily on its transmittance to X-rays, and it is not unique. A fundamental rule is to make the intensity of the light transmitted through the absorber comparable with the strongest scattering intensity of the sample. Different samples have different scattering powers, so absorbers of various thicknesses can be prepared for replacement. However, frequent replacement is inconvenient and affects efficiency. In practice, using a sample with moderate scattering ability, such as glassy carbon, as a reference, the absorber thickness is chosen so that the intensity of the light transmitted through the absorber is comparable with the strongest scattering intensity of the glassy carbon. This chosen thickness generally meets the needs of most samples. The absorber should have two thicknesses with different transmittances to direct light. The thinner thickness should comply with the above rules, while the thicker should have a transmittance to direct light between approximately one-third and two-thirds of that shown by the thinner thickness. By comprehensively considering the component elements of the absorber and its X-ray transmittance, the thickness should ideally be of the order of millimetres or sub-millimetres to facilitate processing.

To determine the relative intensity of the fundamental radiation passing through the sample, attenuation data from two thicknesses of absorber sheets are needed. However, conducting experiments with both thicknesses of absorber sheets separately for each sample and background twice is inconvenient and operationally challenging. Since the beam spot is usually symmetrical in shape and intensity on the detector, the two thicknesses can be combined to form an attenuator. The attenuator is processed into one body with two thicknesses of absorber sheets on the left and right halves. The specific size of the attenuator, like the conventional beamstop, depends on the size of the beam spot and the area where parasitic scattering prevails at the beamstop position (Li *et al.*, 2012 ▸; Gupta *et al.*, 1994 ▸).

During usage, the boundary between the two thicknesses of the absorber sheets on the attenuator is aligned to pass through the beam spot center, with each thickness of the absorber sheet attenuating half of the direct beam. The attenuator constitutes a novel dual-thickness semi-transparent beamstop, which can replace the conventional beamstop with an integrated photodiode. The beamstop can be supported by a slender rod or an X-ray transmitting thin film (*e.g.* polyimide) and fixed to a two-dimensional movable support ring installed inside the end of the camera vacuum tube. By moving the support ring in two dimensions to align it with the direct beam, the beam can be symmetrically distributed over the two thicknesses of the attenuator.

The above design assumes that the beam spot is symmetrical in shape and intensity on the detector. The original design assumes that the beam spot is symmetrical in shape and intensity on the detector. With the development of synchrotron radiation technology, this holds in most cases. However, sometimes, due to structural defects or suboptimal parameter tuning of the light source or optical components, the beam spot may exhibit asymmetry in shape or intensity. In such cases, a single-layer absorber sheet can be used to attenuate the transmitted light before it reaches the detector as shown later in Fig. 2. By observing the shape of the beam spot on the detector and selecting an approximate central line, the integral values on both sides of this central line can be computed, and their ratio, denoted as *c*, can be determined. When the light spot is symmetrical in both shape and intensity, this ratio is *c* = 1; otherwise, *c* ≠ 1. Then, replace the single-layer absorption sheet with the previously mentioned dual-thickness absorption sheet. It is important to ensure that the integration mode and area of the light spot obtained by using the single and double absorbing plates are the same, and the boundary between the two thicknesses of the absorption sheet is aligned with the integral boundary line used to determine the ratio *c*. Thus, equations (6) and (7) can be respectively transformed into equation sets (9) and (10),
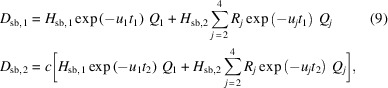

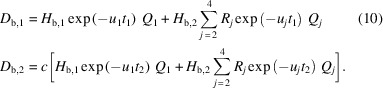
These two equations are applicable regardless of whether the light spot is symmetrical in shape and intensity.

This setup ensures that only one test is needed for each sample and background to collect the transmission beam attenuated by the different thicknesses of the attenuator, facilitating the experiment and improving efficiency to achieve accurate background subtraction.

For specific application examples, see the section below.

## 3. Test at BSRF

To verify the effectiveness and practicality of the new approach, it was applied and compared with the traditional beamstop with an integrated photodiode at the SAXS station on the 1W2A beamline at BSRF (Li *et al.*, 2014 ▸).

### 3.1. Beamline structure

**Figure 1:**
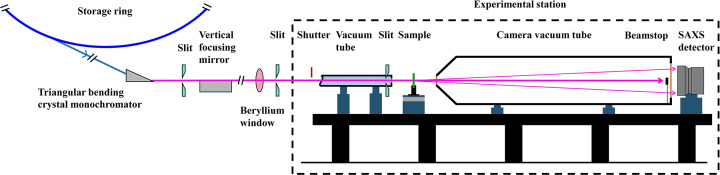
Schematic diagram of SAXS station on 1W2A beamline at BSRF.

The basic structure of the SAXS station on the 1W2A beamline at BSRF is depicted in Fig. 1 ▸. The Beijing Electron Positron Collider (BEPC) storage ring operates at an energy of 2.5 GeV with a beam current of 250 mA. The 1W2A beamline for the SAXS station shares the 1W2 front-end with the 1W2B beamline for the macromolecular station. The 1W2 light source insertion device is a multipole permanent magnet wiggler (14 poles, 7 periods, period length 228 mm).

A triangular bent crystal monochromator is located 20 m from the source, utilizing a Si (111) crystal with a diffraction angle of 2θ = 28.42°. Its horizontal and vertical acceptance ranges are −3.5 to 0 mrad and −0.18 to +0.18 mrad, respectively. Its primary functions include monochromatizing and horizontally focusing the beam (focal length of 10 m), with an outgoing photon energy of 8 keV, corresponding to a wavelength of 1.54 Å.

A rhodium-coated curved cylindrical mirror is placed 2.1 m downstream from the monochromator. Its main function is to vertically focus the beam (with an incident angle of approximately 3 mrad), typically converging to the same focal point as the triangular bending crystal monochromator. The SAXS detector, a Pilatus 1MF, is positioned at this focal point with a beam spot size (FWHM) of approximately 1.2 mm (horizontal) × 0.2 mm (vertical). The photon flux at the sample position can reach up to 5 × 10^11^ photons s^−1^.

The commonly used beamstop is constructed from stainless steel with a length of 12 mm, a width of 6 mm and a thickness of 5 mm. It has a groove shape designed to embed a photodiode.

### 3.2. Harmonic composition

As mentioned above, the 1W2A beamline at BSRF employs a wiggler as the insertion device on the storage ring to generate synchrotron radiation with a low flux of higher-order harmonics. The radiation is monochromated using a Si(111) bent crystal monochromator, and the narrow rocking curve of the crystal’s high-index planes further reduces the flux of higher-order harmonics. The focusing mirror also helps suppress higher-order harmonics. Additionally, the SAXS detector’s efficiency in detecting higher-order harmonics decreases with increasing energy. As a result, the higher-order harmonics are significantly suppressed to meet experimental requirements.

The main harmonics and their content in the incident light can be calculated based on the parameters of the light source and optical elements. There are four main harmonics with serial numbers from 1 to 4 in the incident light, corresponding to the first, third, fourth and fifth orders, with energies of 8 keV, 24 keV, 32 keV and 40 keV, respectively.

### 3.3. New beamstop design

**Figure 2:**
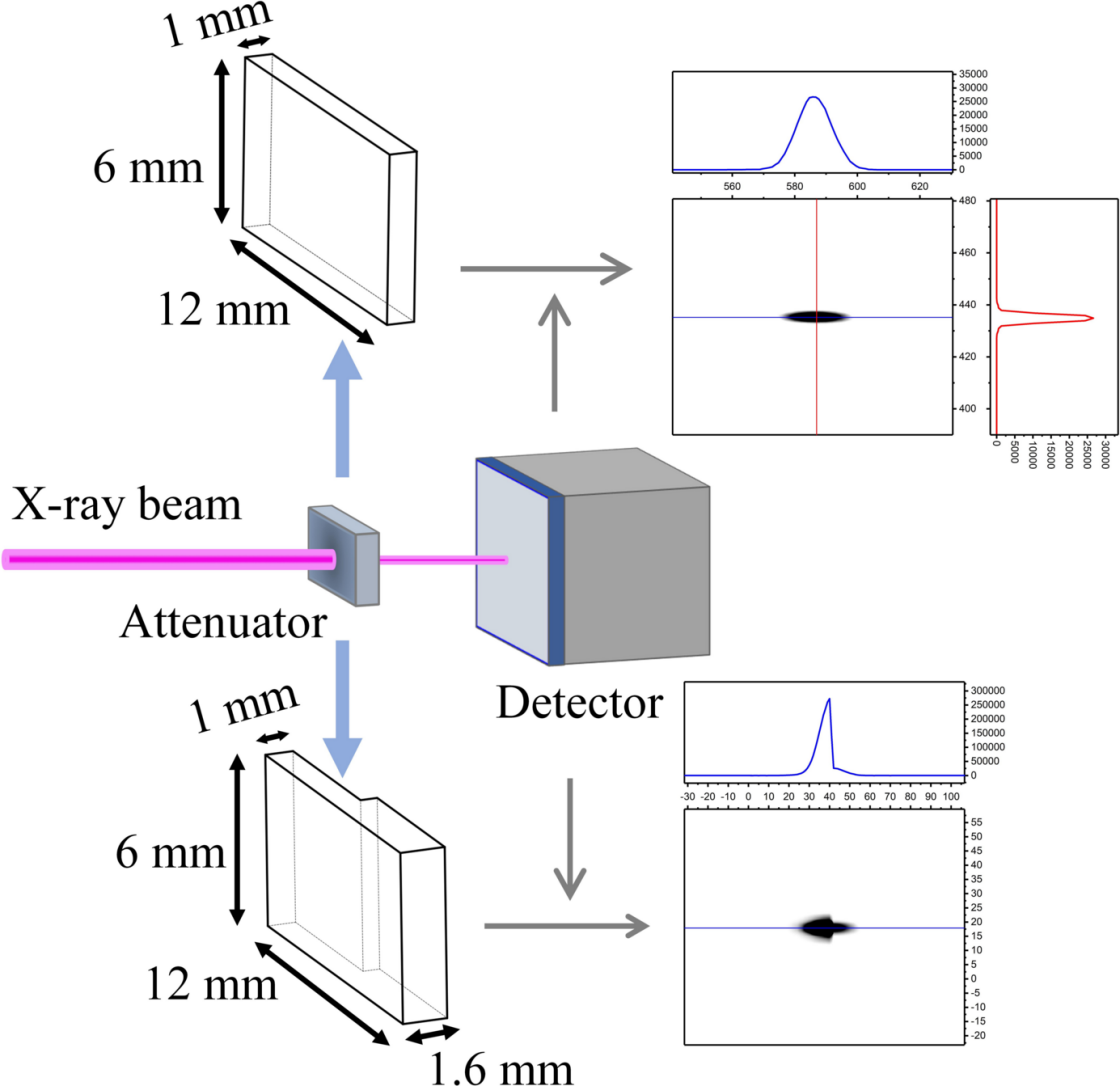
Attenuators with single and double thicknesses, along with the corresponding beam spot images on the Pilatus 1MF detector at the SAXS station on 1W2A beamline at BSRF.

According to the selection rules mentioned in Section 2 and using glassy carbon as a reference sample, pure aluminium (Al) absorber sheets of 1.0 mm and 1.6 mm thickness can be used for machining the attenuator. Similar to the conventional beamstop currently used at this station, a rectangular aluminium attenuator is machined with dimensions of 12 mm (length) × 6 mm (width) × 1.0 mm/1.6 mm (thickness), as shown in Fig. 2 ▸. The attenuator thickness varies, with one half being 1.0 mm and the other half being 1.6 mm. The attenuator is supported by a slender rod or an X-ray transmitting thin film (*e.g.* polyimide), fixed to the same two-dimensional movable support mechanism as the conventional beamstop, and aligned with the transmission beam. Once the transmission beam spot is symmetrically distributed on the attenuator of two thicknesses, the experiment can commence. The beam spot passing through the attenuator is shown in Fig. 2 ▸.

The linear absorption coefficients of Al for the fundamental (8 keV), third (24 keV), fourth (32 keV) and fifth (40 keV) harmonics are 13.58 mm^−1^, 0.49 mm^−1^, 0.27 mm^−1^ and 0.15 mm^−1^, respectively (Hubbell & Seltzer, 1995 ▸). Table 1 ▸ lists the harmonic composition and corresponding transmittance through several typical samples with optimal thickness for 8 keV fundamental radiation, as well as through Al absorber sheets with thicknesses of 1.0 mm and 1.6 mm. Table 2 ▸ provides examples of harmonic relative flux (photons s^−1^) in the direct beam, through a glassy carbon (C) sample with an optimal thickness of 1.28 mm for 8 keV fundamental radiation, through Al absorber sheets with thicknesses of 1.0 mm and 1.6 mm, and as received by the Pilatus 1MF detector, assuming a total incident flux at the sample of 5 × 10^11^ photons s^−1^.

### 3.4. Performance test

SAXS tests were conducted on three typical samples at this station using both the new and old beamstops, which are supported by a slender rod and a polyimide thin film with a small hole, respectively. The detector-to-sample distance was set to 1671 mm. The samples tested include a standard sample of glassy carbon SRM3600, sourced from the National Institute of Standards and Technology (NIST) (Allen *et al.*, 2017 ▸; Small & Choquette, 2016 ▸). It is a square-shaped solid piece with a side length of 10 mm and a thickness of 1.055 mm, directly applicable for testing with an exposure time of 30 s, using air as the background with an additional background measurement taken with an exposure time of 20 s. The second sample was a lump of anthracite, obtained from Jincheng Temple River Coal Mine in Shanxi Province, China. This sample was ground into powder and filled into a circular transparent sample chamber with a diameter of 10 mm and a thickness of 1 mm. The testing exposure time was 5 s, and an empty sample chamber was used as the background with an exposure time of 20 s. The third sample was a colloidal silica solution, purchased from Hangzhou Hengge Nanotechnology Co. Ltd, with sol particles approximately 30 nm in diameter and a mass concentration of 0.04 wt%. This solution was loaded into a 1 mm-thick transparent sample cell and tested with an exposure time of 30 s, using the cell filled with water as the background with an exposure time of 60 s.

**Figure 3:**
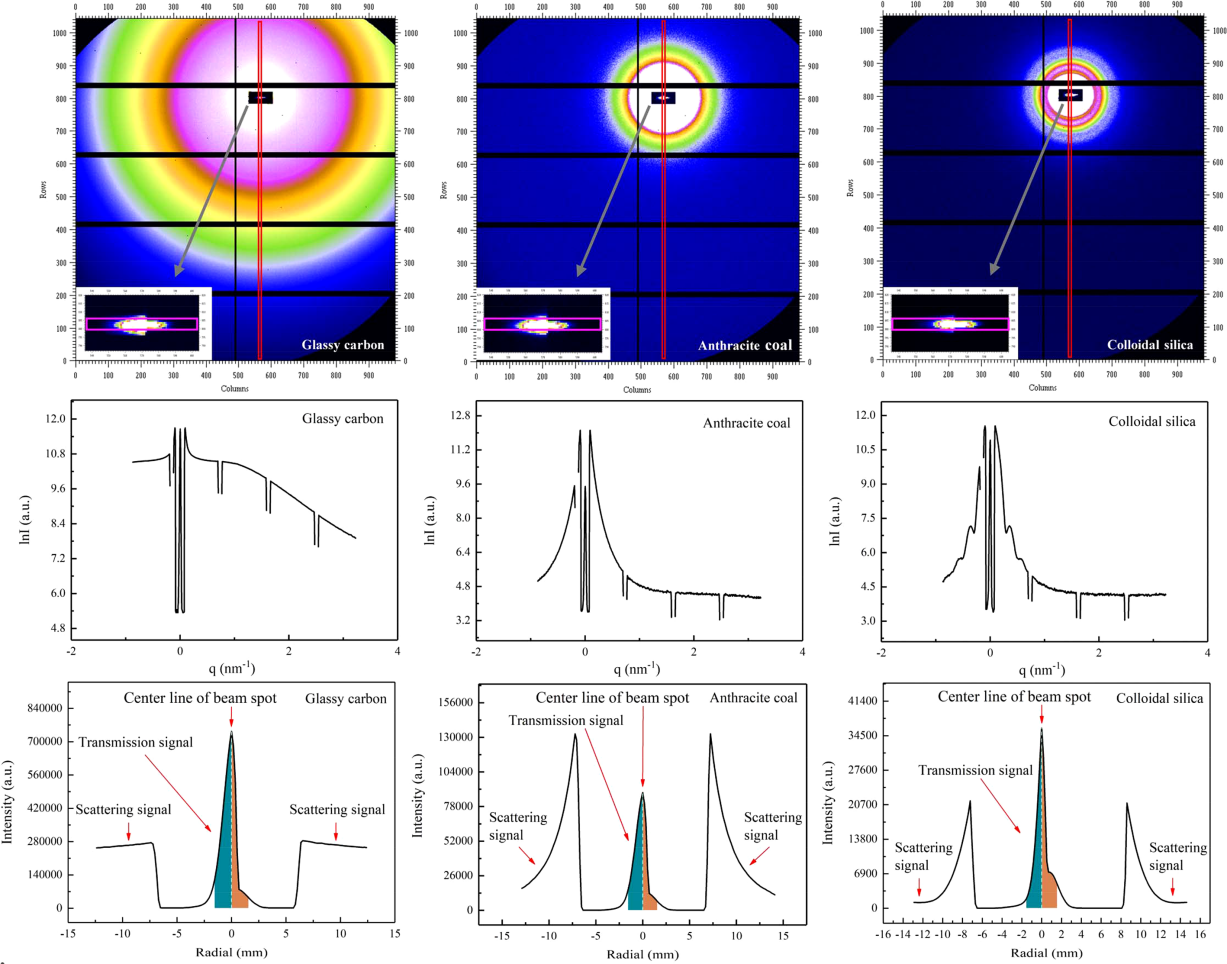
Experimental results at the SAXS station on 1W2A beamline at BSRF, showing two-dimensional images (without time normalization) of scattered signals and amplified images (after time normalization) of transmitted signals after passing through the new beamstop collected by a Pilatus 1MF detector. The corresponding one-dimensional scattering curves are derived by vertical rectangular integration in the red rectangle zone on the scattered signal images, and transmission curves are derived by horizontal rectangular integration in the pink rectangle zone on the transmitted signal images.

The scattering signals and attenuated transmitted signals are collected simultaneously by a Pilatus 1MF detector. Fig. 3 ▸ shows the experimental results, including two-dimensional images (without time normalization) of scattered signals, amplified images (after time normalization) of transmitted signals after passing through the new beamstop, corresponding one-dimensional scattering curves derived by vertical rectangular integration in the red rectangle zone on the scattered signal images, and transmission curves derived by horizontal rectangular integration in the pink rectangle zone on the transmitted signal images. The integration was performed using *Fit2d* software (Hammersley, 2016 ▸). All the samples showed strong isotropic scattering, with scattering intensity decreasing as the angle increases. The horizontal and vertical black stripes on the images, as well as the ‘dips’ in intensity on the one-dimensional curves, correspond to dead zones formed by the seams between the sensitive modules of the detector.

The ‘shadow’ of the beamstop is visible on the two-dimensional images, with bright spots appearing within the black shadows, indicating that the transmission beam was not completely blocked by the beam attenuator but was attenuated and received by the detector. Consequently, three peaks are visible on the one-dimensional curve (excluding peaks formed by the detector’s dead zones). The middle peak represents the attenuated transmission beam signals, while the peaks on the sides represent the strongest scattering signals of the sample.

As shown in Fig. 2 ▸, synchrotron radiation beam spots are generally larger in horizontal size and divergence than in vertical size and divergence. The spatial resolution of scattering images is better in the vertical direction than in the horizontal direction. Therefore, the integration of the scattered signals is usually performed in the vertical direction. Nevertheless, because the horizontal size of the beam spot is larger than the vertical size, the transmitted signal integration needs to be carried out in the horizontal direction to increase statistics.

The specific integration process to determine the relative intensity of the transmission beam consists of two steps. Firstly, the transmission beam image is horizontally integrated with an integration width of 3σ_v_ = 0.51 mm, where σ_v_ is derived from the vertical size of the beam spot in Fig. 2 ▸. This integration results in a one-dimensional scattering curve. Next, integration is performed on both sides of the central line (white dashed line) of the beam spot in Fig. 3 ▸, with an integration width of 1.5σ_h_ = 1.53 mm, where σ_h_ is derived from the horizontal size of the beam spot in Fig. 2 ▸. The final integration results on the left and right sides correspond to the relative intensities of the transmission beam attenuated by aluminium absorber sheets of thickness 1.0 mm and 1.6 mm, respectively.

To subtract the background scattering and obtain the pure sample scattering, it is essential to test and integrate the background of each sample under the same conditions as the sample itself.

**Figure 4:**
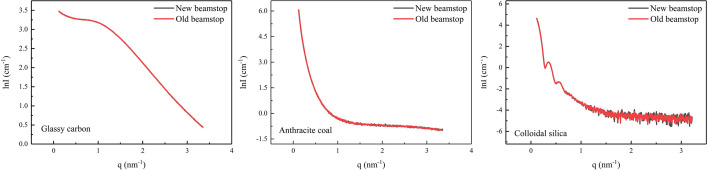
Comparison of background subtraction results of three typical samples tested using the new and old beamstops, respectively, at the SAXS station on 1W2A beamline at BSRF.

The equation sets (9) and (10) can be solved to obtain *H*_sb,1_ and *H*_b,1_, where *c* ≃ 1 as demonstrated in Fig. 2 ▸. Using these, the normalization factor *K*_sb_/*K*_b_ can be calculated according to equation (8), which can then be substituted into equation (1) to perform background subtraction. Fig. 4 ▸ shows a comparison of the background subtraction results for the three samples tested with the new beamstop and the conventional beamstop with an integrated photodiode. The background subtraction calculation is performed using the *S* software (Li, 2013 ▸). The results from both methods are in good agreement under routine longer exposure times. Similar background subtraction results can be achieved by slightly adjusting the exposure time or the thickness combination of absorber pieces in the new beamstop, which are not elaborated on here.

**Figure 5:**
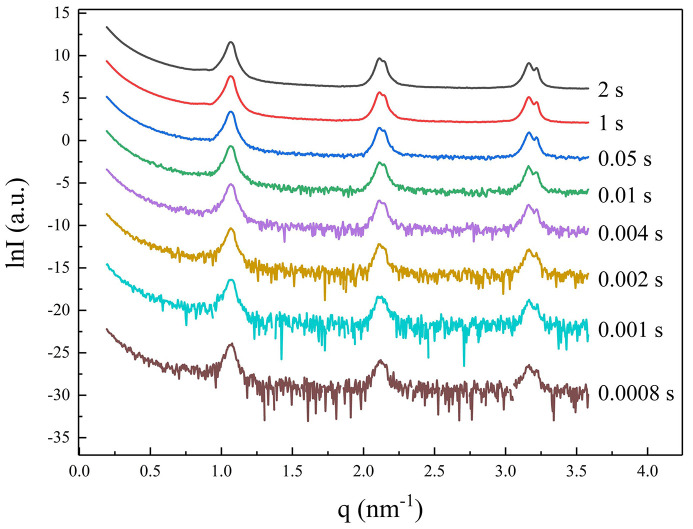
Scattering curves of silver behenate sample with different exposure times at the SAXS station on 1W2A beamline at BSRF.

It is evident that the systematic errors of the traditional method, which uses two different systems to measure scattered light and transmitted light, are not easy to detect and can be neglected during the conventional longer exposure times that users are familiar with. To test the hypothesis on improved synchronization, a strongly scattering silver behenate sample (with strong Bragg reflections at low *q*) was measured with different exposure times, and the results are shown in Fig. 5 ▸ and Table 3 ▸. It can be seen that, when the exposure time is short, such as ≤4 ms, the response speed of the photodiode cannot keep up with the detection speed of the SAXS detector at this station. This problem does not exist in the new approach because it simultaneously detects both scattering and transmission signals using the same detector, ensuring absolute synchronization. Therefore, this approach is particularly suitable for fast time-resolved SAXS experiments using synchrotron radiation.

## 4. Prospect at HEPS

### 4.1. Beamline

Benefiting from the high storage ring electron beam energy and low emittance of the High Energy Photon Source (HEPS), the pink beam mode of the pink SAXS station at HEPS can directly use the quasi-monochromatic central cone radiation of the insertion device undulator without the need for a monochromator or a focusing mirror (Li *et al.*, 2021 ▸). The beamline structure is very simple, providing a high-flux, highly stable beam with adjustable energy, typically optimized at 12 keV. The station will have a high photon flux greater than 10^15^ photons s^−1^, making it particularly suitable for conducting fast (millisecond, microsecond or even sub-microsecond) time-resolved experiments such as dust explosion studies (Wu *et al.*, 2024 ▸). This will be one of the main scientific targets of the upcoming fourth-generation synchrotron radiation light source HEPS. The approach proposed in this contribution is therefore of great significance in making full use of the advantages of HEPS.

### 4.2. Beamstop

Based on the design parameters of the light source and beamline optical elements (Li *et al.*, 2021 ▸), the harmonic composition of the incident light at this station can be calculated, as shown in Table 4 ▸. Similarly, it can be approximated that the flux ratio between higher-order harmonics remains essentially unchanged before and after passing through the sample measured by the fundamental radiation of 12 keV, as shown in Table 5 ▸. In this case, the approach proposed in this study becomes applicable. For instance, the attenuator can be machined with titanium sheets (thickness of 0.50 mm and 0.55 mm) or silicon carbide sheets (thickness of 3.00 mm and 3.30 mm), which have melting points of 1668°C and 2000°C, respectively, and exhibit good mechanical and chemical stability at high temperatures. The specific dimensions of the attenuator depend on the size of the beam spot and the area where parasitic scattering prevails at the beamstop position near the detector.

As a typical case, with the sample positioned 50 m from the light source and the detector 5 m downstream of the sample, a 0.5 mm × 0.5 mm beam spot can be obtained on the detector, yielding symmetrical and statistically sound scattering images. According to optical design (Li *et al.*, 2012 ▸, 2021 ▸), the size of the attenuator near the detector needs to be at least 2 mm × 2 mm, which can be safely implemented using the new approach. The specific dimensions of the blocking device will need to be adjusted based on the actual beam and parasitic scattering conditions at that time. Several beamstops with similar structures, and different materials and specifications can be prepared for replacement.

## 5. Discussion

### 5.1. Conventional beamstops

In SAXS, commonly used methods for measuring the intensity of transmitted direct light include early ionization chambers and later photodiodes. These methods employ different detectors and electronic control systems to separately measure transmission and scattering signals. However, achieving true synchronization is challenging, as the counting rates, response speeds and detection efficiencies of the two systems differ. This discrepancy leads to errors in intensity measurements, especially when the signals are weak or the exposure time is short, as exampled in Fig. 5 ▸ and Table 3 ▸.

Some researchers have developed a semi-transparent beamstop that uses a single-thickness absorber to attenuate the direct light passing through the sample, suitable for ideal pure monochromatic beams (Lyngsø & Pedersen, 2021 ▸). This allows the direct light to be received by the scattering signal detector, which then back-calculates the intensity of the fundamental radiation passing through the sample. This method achieves true synchronization between the scattering signal and the transmission signal measurements. However, any light source inevitably generates higher-order harmonics alongside fundamental radiation. By optimizing the light source and optical components, higher-order harmonics can be suppressed to an acceptable level. As shown in Table 2 ▸, the absorber significantly attenuates the fundamental wave. Although the higher-order harmonics change little after passing through the sample and remain negligible compared with the fundamental wave, they become non-negligible after passing through the absorber. In the single-thickness semi-transparent beamstop method, the contribution of higher-order harmonics cannot be separated, introducing a certain error and limiting its application.

### 5.2. New beamstop

To address these shortcomings and enhance the accuracy of background subtraction, this study develops a gradient attenuation approach to measure the transmission beam intensity and subtract the background of scattering intensity. This method replaces a beamstop made of high-*Z* elements with a beam attenuator made of thin or low-*Z* elements that absorb the direct beam partially. This allows the detector to simultaneously detect both scattering and transmission signals, achieving true synchronization in measurement, beneficial for fast time-resolved experiments. It also replaces the single-thickness absorber sheet beamstop with an attenuator consisting of two thicknesses of absorber sheets arranged side by side. This allows for the elimination of the contribution of higher-order harmonics during the inverse derivation of the transmission intensity of fundamental radiation. Moreover, even for an ideal pure single-energy beam, dual-thickness semi-transparent beamstops are still applicable. This approach is not sensitive to visible light, so no special shielding device is required.

### 5.3. Higher-order harmonic ratio hypothesis

The new approach assumes that the flux ratio between higher-order harmonics remains essentially unchanged before and after passing through the samples. This assumption is used to determine *R*_*j*_ in equation (4), which depends on the X-ray energy and the sample. Additionally, the absorption edge energy of the sample should not coincide with the energy of the higher harmonics. It is well known that there exists an optimal sample thickness for SAXS as *t*_opt_ = 1/μ to obtain the best statistical scattering data (Meyers, 2001 ▸), where μ represents the linear absorption coefficient of the sample. In practice, the sample thickness can be within a range to ensure that the transmittance is within one-third to two-thirds. From Tables 1 ▸ and 2 ▸, it can be seen that samples suitable for experiments using fundamental radiation (*i.e.* first harmonic) usually exhibit low absorption and high transmittance for higher-order harmonics, and the ratios between higher-order harmonics after passing through the sample can be approximately considered unchanged. Therefore, the higher-order harmonics can be integrated into one unknown variable, in addition to the fundamental unknown variable, resulting in two unknowns. This rule likely applies to most synchrotron radiation SAXS stations, validating the hypothesis.

### 5.4. Incident light flux, detector pixel size and count rate

As mentioned earlier, the absorber is not used to attenuate the incident light but rather to attenuate the direct transmitted light through the sample to a level comparable with the scattering signals of the sample so that the transmission signals can be received by the scattering signal detector. The selection of the material and thickness of the absorber are not directly related to the incident light flux, detector pixel size and count rate. The incident light flux affects the exposure time, with higher flux resulting in shorter exposure times, beneficial for time-resolved experiments. X-ray diffraction (XRD) spectra typically exhibit sharp diffraction peaks (Lamas *et al.*, 2017 ▸), requiring the smallest possible detector pixel size to improve spatial resolution. Unlike XRD, SAXS spectra usually appear as a curve similar to a ‘backrest chair’ and do not require high pixel resolution. For example, Pilatus detectors with pixel sizes of 172 µm have been widely used for SAXS experiments with incident flux below 10^13^ photons s^−1^. However, the detector’s count rate needs to match the incident light flux to meet different time resolution requirements. For instance, at the SAXS station at the BSRF, the incident light flux is approximately at the level of 10^11^ photons s^−1^, and photon-counting detectors (such as Pilatus) have proven suitable. In contrast, the forthcoming pink SAXS station at HEPS with a light flux up to 10^15^ photons s^−1^ will require an integrating detector for the pink beam mode; otherwise, the incident light will have to be attenuated to use a photon-counting detector. These are key considerations for the design of experimental stations and are beyond the scope of this contribution.

### 5.5. Thermal load

For high-flux beams, such as the pink beam mode of the SAXS station at HEPS, where the flux will reach over 10^15^ photons s^−1^, special attention must indeed be paid to thermal load issues. Such high flux cannot be addressed with traditional thinking and approaches (Ko *et al.*, 2023 ▸). High flux significantly shortens exposure time, making it particularly suitable for time-resolved experiments. Consequently, the speed of control (such as shutters) and detection systems (such as detectors) must match this flux. Therefore, the thermal load must be borne by the optical elements upstream of the sample, rather than by the beamstop downstream, otherwise the sample will be damaged or even burned. Nevertheless, the beamstop should be made of materials with high time and temperature stability.

### 5.6. Beam spot size

The beamstop can be prepared with different sizes according to different beam spot sizes and parasitic scattering levels limited by different slit combinations. It might seem that a larger beamstop is always better, but, even for beamlines with focusing modes, the beamstop is much larger than the beam spot and pixel size at the detector (Gupta *et al.*, 1994 ▸; Li *et al.*, 2012 ▸), and modern machining technology fully meets the micromachining requirements. Beamstops of at least 1 mm × 1 mm and above can be used in the new approach. Even though a smaller beamstop might be more challenging to process, the main limitation is compatibility with detectors having smaller pixel sizes, which deserves further study.

### 5.7. Beam spot support mode

The conventional beamstop with a photodiode uses a hollow rod to support the beamstop and its signal cable (Desjardins *et al.*, 2021 ▸; Englich *et al.*, 2011 ▸). The new beamstop has no signal cable and can be supported by a rod or a piece of light-transmitting film. The support rod does not produce additional scattering but can form a shadow, which is not conducive to the complete display of the anisotropic image. Thin films can produce additional scattering and even diffraction, increasing the background, which is unfavorable for improving the signal-to-noise ratio, especially for weakly scattered signals. Therefore, the support mode should be selected according to the specific sample. Many polymer films can produce diffraction rings under direct irradiation of strong light, which are difficult to deduct. When the beamstop is carried by a film, the beamstop needs to be glued to the back of the film so that the direct light passes through the film first and then through the beamstop, blocking the film’s diffraction ring. Alternatively, a small hole can be made in the film, and the beamstop can be attached to the hole’s periphery to avoid direct light irradiation of the film.

### 5.8. Advantage

The attenuator in this new approach is simple in structure, easy to process and convenient to use. This approach omits the special detection system for the transmission beam, simplifies the instrument structure, saves the cost of instrument construction and maintenance, improves the accuracy of background subtraction, solves the background subtraction problem of fast time-resolution experiments, and is expected to apply to many SAXS instruments and other techniques such as X-ray photon correlation spectroscopy (Shpyrko, 2014 ▸), X-ray cross-correlations analysis (Schroer *et al.*, 2015 ▸) and ptychographic coherent X-ray diffractive imaging (Reinhardt *et al.*, 2017 ▸; Wilke *et al.*, 2013 ▸). However, this approach also has its disadvantages, such as the absorption limit of the sample not coinciding with the fundamental radiation energy of the incident light. For this type of sample, it is necessary to replace the absorber sheet with another material.

## 6. Summary

In a SAXS experiment, while the scattered light is measured, the transmitted light needs to be measured and blocked to subtract the background from the scattering intensity and protect the detector. The commonly used method is to integrate a photodiode with a beamstop to measure and block the transmitted light. However, they cannot provide sufficient dynamic range and sensitivity under the conditions of strong scattering signal and short exposure time. The advent of the single-thickness semi-transparent beamstop allows the scattering signal detector to receive the attenuated transmitted signal, achieving true synchronization between the scattering and transmission signal measurements but cannot peel off the contribution of high-order harmonics.

This study develops a novel dual-thickness semi-transparent beamstop that cannot only cover the application range of traditional methods but also effectively overcome the aforementioned drawbacks and extend to areas where traditional methods are not applicable, thereby expanding the functionality and application of SAXS. It processes an attenuator into two thicknesses, half on each side. The transmitted light is attenuated in gradient and received by the scattered light detector. The share of high-order harmonics is stripped off, and the transmitted fundamental radiation intensity is inversely deduced to subtract the background. The specific steps of this new approach include determining the harmonic composition of the incident beam, selecting absorber materials, designing and fabricating the beam attenuator, testing samples, and their backgrounds, integrating signals, deducing the transmission beam intensity, and subtracting the background from scattering intensity.

## Figures and Tables

**Table 1:** Harmonic composition in the incident beam, corresponding transmittance through several typical samples with optimal thickness for 8 keV fundamental radiation, and Al absorber sheet with respective thicknesses of 1.0 mm and 1.6 mm, and quantum counting efficiency of Pilatus 1MF detector at the SAXS station on 1W2A beamline at BSRF

Light source			Wiggler (14 poles, 7 periods, period length 228 mm)			
Monochromator crystal reflecting surface			Si (111)	Si (333)	Si (444)	Si (555)
Harmonic serial number			1	2	3	4
Harmonic order			First	Third	Fourth	Fifth
Energy (keV)			8	24	32	40
Transmittance of typical samples with optimal thickness for 8 keV fundamental radiation	C	1.28 mm	0.36	0.93	0.94	0.95
	H_2_O	0.96 mm	0.36	0.95	0.96	0.97
	Al	73 µm	0.36	0.96	0.98	0.98
	Fe	4.2 µm	0.36	0.95	0.97	0.98
Transmittance of absorber with different thickness	Al	1.0 mm	1.26 × 10^−6^	6.12 × 10^−1^	7.61 × 10^−1^	8.58 × 10^−1^
	Al	1.6 mm	3.64 × 10^−10^	4.56 × 10^−1^	6.46 × 10^−1^	7.82 × 10^−1^
Quantum counting efficiency of Pilatus 1 MF			0.972	0.159	0.066	0.033

**Table 2:** Harmonic relative flux (photons s^−1^) in direct beam, through sample glass carbon (C) with optimal thickness of 1.28 mm for 8 keV fundamental radiation, through Al absorber sheet with respective thicknesses of 1.0 mm/1.6 mm, and received by Pilatus 1MF detector, supposing a total incident flux at sample of 5 × 10^11^ photons s^−1^ at the SAXS station on 1W2A beamline at BSRF

Energy (keV)	8	24	32	40
Incident beam	4.99 × 10^11^	4.13 × 10^8^	9.37 × 10^6^	2.25 × 10^5^
Through sample C 1.28 mm	1.84 × 10^11^	3.86 × 10^8^	8.93 × 10^6^	2.16 × 10^5^
Through absorber Al 1.0 mm/1.6 mm	1.2 × 10^5^/3.3 × 10^1^	1.2 × 10^8^/8.8 × 10^7^	3.4 × 10^6^/2.9 × 10^6^	9.3 × 10^5^/8.5 × 10^5^
Received by detector 1.0 mm/1.6 mm	1.1 × 10^5^/3.3 × 10^1^	2.6 × 10^7^/1.9 × 10^7^	3.2 × 10^5^/2.7 × 10^5^	4.6 × 10^3^/4.1 × 10^3^

**Table 3:** Comparison of transmitted signal response during SAXS experiments on a silver behenate sample with different exposure times, using new and old approaches at the SAXS station on 1W2A beamline at BSRF

Exposure time (s)	Transmitted signal response	
	Photodiode counting	Maximum intensity on detector
2	1108857	147347
1	55344	73633
0.05	2503	3772
0.01	320	776
0.004	0	299
0.002	0	165
0.001	0	78
0.0008	0	75

**Table 4:** Harmonic composition in incident beam and corresponding transmittance through several typical samples with optimal thickness for 12 keV fundamental radiation, and titanium absorber sheets with respective thicknesses of 0.50 mm and 0.55 mm, at the pink beam SAXS station at HEPS

Light source			Undulator (IAU25)				
Harmonic serial number			1	2	3	4	5
Harmonic order			First	Second	Third	Fourth	Fifth
Energy (keV)			12	24	36	48	60
Transmittance of typical samples with optimal thickness for 12 keV fundamental radiation	C	4.2 mm	0.37	0.80	0.85	0.87	0.88
	H_2_O	3.3 mm	0.37	0.84	0.90	0.93	0.93
	Al	0.25 mm	0.37	0.88	0.95	0.97	0.98
	Fe	12.6 µm	0.37	0.87	0.95	0.98	0.99
Transmittance of titanium absorber sheets	Ti	0.50 mm	1.55 × 10^−6^	1.80 × 10^−1^	4.66 × 10^−1^	7.45 × 10^−1^	8.40 × 10^−1^
	Ti	0.55 mm	4.08 × 10^−7^	1.52 × 10^−1^	4.32 × 10^−1^	7.24 × 10-1	8.26 × 10^−1^

**Table 5:** Harmonic relative flux (photons s^−1^) in direct beam, through sample glass carbon (C) with optimal thickness of 4.2 mm for 12 keV fundamental radiation, through titanium (Ti) absorber sheet with respective thicknesses of 0.5 mm/0.55 mm, supposing a total incident flux at the sample of 3.21 × 10^15^ photons s^−1^ at the pink beam SAXS station at HEPS

Energy (keV)	12	24	36	48	60
Incident beam	3.21 × 10^15^	4.17 × 10^8^	6.07 × 10^7^	7.63 × 10^4^	1.64 × 10^4^
Through sample C 4.2 mm	1.18 × 10^15^	3.32 × 10^8^	5.16 × 10^7^	6.65 × 10^4^	1.44 × 10^4^
Through absorber Ti 0.5 mm / 0.55 mm	1.8 × 10^9^ / 4.8 × 10^8^	6.0 × 10^7^ / 5.0 × 10^7^	2.4 × 10^7^ / 2.2 × 10^7^	5.0 × 10^4^ / 4.8 × 10^4^	1.2 × 10^4^ / 1.1 × 10^4^

## Data Availability

The authors confirm that the data supporting the findings of this study are available within the article and its supplementary materials.
